# Semi-automatic tumor segmentation of rectal cancer based on functional magnetic resonance imaging

**DOI:** 10.1016/j.phro.2022.05.001

**Published:** 2022-05-11

**Authors:** Franziska Knuth, Aurora R. Groendahl, René M. Winter, Turid Torheim, Anne Negård, Stein Harald Holmedal, Kine Mari Bakke, Sebastian Meltzer, Cecilia M. Futsæther, Kathrine R. Redalen

**Affiliations:** a Department of Physics, Norwegian University of Science and Technology, Høgskoleringen 5, 7491 Trondheim, Norway; b Faculty of Science and Technology, Norwegian University of Life Sciences, Drøbakveien 31, 1432 Ås, Norway; c Department of Informatics, University of Oslo, Gaustadalléen 23 B, 0373 Oslo, Norway; d Institute for Cancer Genetics and Informatics, Oslo University Hospital, Ullernchausséen 64, 0379 Oslo, Norway; e Department of Radiology, Akershus University Hospital, Sykehusveien 25, 1478 Nordbyhagen, Norway; f Institute of Clinical Medicine, University of Oslo, Kirkeveien 166, 0450 Oslo, Norway; g Department of Oncology, Akershus University Hospital, Sykehusveien 25, 1478 Nordbyhagen, Norway

**Keywords:** ADA, Adaptive boosting, DICE, Sørensen-Dice similarity coefficient, DME, Dynamic multi echo, DW, Diffusion weighted, IQR, Interquartile range, LDA, Linear discriminant analysis, MED, Median, MRI, Magnetic resonance imaging, MSD, Mean symmetric surface distance, SVM, Support vector machines, QDA, Quadratic discriminant analysis

## Abstract

**Background and purpose:**

Tumor delineation is required both for radiotherapy planning and quantitative imaging biomarker purposes. It is a manual, time- and labor-intensive process prone to inter- and intraobserver variations. Semi or fully automatic segmentation could provide better efficiency and consistency. This study aimed to investigate the influence of including and combining functional with anatomical magnetic resonance imaging (MRI) sequences on the quality of automatic segmentations.

**Materials and methods:**

T2-weighted (T2w), diffusion weighted, multi-echo T2*-weighted, and contrast enhanced dynamic multi-echo (DME) MR images of eighty-one patients with rectal cancer were used in the analysis. Four classical machine learning algorithms; adaptive boosting (ADA), linear and quadratic discriminant analysis and support vector machines, were trained for automatic segmentation of tumor and normal tissue using different combinations of the MR images as input, followed by semi-automatic morphological post-processing. Manual delineations from two experts served as ground truth. The Sørensen-Dice similarity coefficient (DICE) and mean symmetric surface distance (MSD) were used as performance metric in leave-one-out cross validation.

**Results:**

Using T2w images alone, ADA outperformed the other algorithms, yielding a median per patient DICE of 0.67 and MSD of 3.6 mm. The performance improved when functional images were added and was highest for models based on either T2w and DME images (DICE: 0.72, MSD: 2.7 mm) or all four MRI sequences (DICE: 0.72, MSD: 2.5 mm).

**Conclusion:**

Machine learning models using functional MRI, in particular DME, have the potential to improve automatic segmentation of rectal cancer relative to models using T2w MRI alone.

## Highlights

• Machine learning on magnetic resonance images (MRI) was used for tumor segmentation.
• Voxelwise machine learning with morphological post-processing achieved good segmentation results.
• Combining T2-weighted with functional MRI improved semi-automatic tumor segmentation.
• Dynamic contrast enhanced MRI was the most valuable functional MRI information.
• Tumor volume and interobserver variation were linked to measured segmentation quality.

## Graphical abstract

**Figure:**
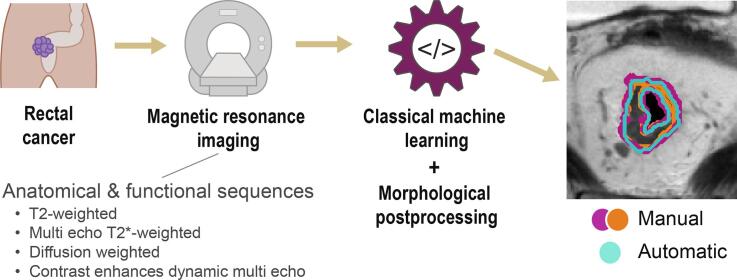

## 1 Introduction

Tumor volume definition is an integral part of radiotherapy planning. Increasingly, it is also required for quantitative image biomarker purposes [1] and plan-of-the-day adaptive radiotherapy [2]. The current gold standard for tumor volume definition is manual delineation, which is a time- and labor-intensive process. It has also been entitled the weakest link in radiotherapy planning [3], in part due to inter- and intraobserver variations. High interobserver variations have been reported for several cancer types including rectal cancer [4], [5], [6], which was the 8th most common cancer type in 2020 and contributed 3.8% of all new reported cases globally [7].

Radiotherapy planning for rectal cancer is most often based on computer tomography (CT), but there is a trend to increase the use of magnetic resonance imaging (MRI) [8]. The gold standard for local tumor staging already includes MRI. Anatomical T2-weighted (T2w) images offer superior soft tissue contrast compared to CT. In addition, functional MRI sequences can provide insights into biological properties of the imaged tissue. A commonly used functional MRI method, that also is recommended to include in the staging protocol [9], is diffusion weighted (DW) MRI, where the image contrast depends on the microscopic mobility of water and gives insight into tissue structure and perfusion [10], [11]. Two functional MRI sequences that are more exploratory in rectal cancer are T1-weighted dynamic contract enhanced (DCE) MRI and T2*-weighted (T2*w) MRI. DCE MRI requires injection of a contrast agent and repeated imaging over several minutes, and depicts tissue vascularity and permeability of the vessels [12]. The method has shown to be promising for rectal cancer [13], although it is not part of the current international guidelines. Multi echo T2*w imaging is a method that visualizes endogenous paramagnetic deoxyhemoglobin, which in breast cancer has been shown to correlate to tumor hypoxia [14]. In rectal cancer, the method has recently shown potential to provide a useful quantitative biomarker [15].

To date, different semi- and fully automatic segmentation methods based on various image modalities have been developed for rectal cancer [5], [16], [17], [18], [19]. Soomro et al. compared different level set methods using T2w MRI to segment the entire colorectal region [16]. Heeswijk et al. presented a region growing based method using DW MRI as input [17]. Ciernik et al. used a similar method with positron emission tomography (PET) images [18]. Bisgaard et al. used thresholding based on DW MRI where T2w MRI identified the initial region of interest [19]. Another approach utilizing supervoxel segmentation was explored by Irving et al., based on DCE MRI [5]. All these studies relied on a single image type and modality as input. To our knowledge, there is a lack of studies systematically investigating the combined use of different, multi-sequence images for tumor segmentation. Recognizing that T2w MRI and the various functional MRI methods (DWI, contrast-based MRI, T2*w-MRI) provide different and unique image contrasts, our hypothesis was that inclusion of one or several of the functional MRI sequences would improve segmentation performance compared to using T2w MRI alone as input to the segmentation algorithm.

The aim of this study was to examine the influence of including and combining anatomical and multi-sequence functional MRI sequences on the quality of semi- or fully automatic segmentations of rectal cancer.

## 2 Materials and methods

### 2.1 Patients

The patient data in this study was from a prospective observational trial (OxyTarget, clinicaltrials.gov no. NCT01816607) enrolling patients with suspected rectal cancer between October 2013 and December 2017. Eligible participants had histologically confirmed rectal adenocarcinoma, were older than 18 years, and had no prior rectal cancer treatment. Participants were enrolled consecutively. OxyTarget included a total of 192 patients. In the current study, data from 81 patients was analyzed. These patients had successful image acquisition with adequate image quality for analysis, without artifacts nor other distortions. Other reasons for exclusion were incomplete data sets. Further details regarding exclusion criteria have been described previously [20]. The analyzed patient cohort consisted of 53 men and 28 women with a median age of 64 years. Based on MRI, the tumors were staged as T2/T3/T4 with 12/41/28 cases respectively. Further patient statistics were summarized in Table 1. For all patients, written informed consent was obtained and the study was performed in accordance with the Helsinki Declaration. Approval was obtained from the Institutional Review Board and the Regional Committee for Medical and Health Research Ethics.

**Table 1:** Overview of patient characteristics.

Age / years	Median	64
	Range	41–88
Sex	Male	53 (65%)
	Female	28 (35%)
Tumor site	Rectum	76 (94%)
	Rectosigmoid	5 (6%)
Tumor stage	T2	12
	T3	41
	T4	28
Nodal stage	N0	35
	N1	28
	N2	17
	N3	1
Tumor volume / cm^3^	Median	28.7
	Range	2.1–168.2

### 2.2 Magnetic resonance imaging and manual delineation

MRI was performed on a Philips Achieva 1.5 T system (Philips Healthcare, Best, The Netherlands) to acquire routine and study specific images. In addition to T2w images, an extended DW sequence with seven b-values of b = 0, 25, 50, 100, 500, 1000 and 1300 s/mm^2^ was obtained. A static T2*w MRI sequence with five echo times (TE) = 4.6, 13.8, 23.0, 32.2 and 41.4 ms and a dynamic multi echo (DME) contrast MRI sequence with three echoes with TE = 4.6, 13.9 and 23.2 ms were collected. The latter was acquired using a split dynamic acquisition previously described in [21] and a bolus injection of 0.2 ml/kg body weight of Dotarem® (279.3 mg/ml, Guerbert Roissy, France), directly followed by a 20 ml saline solution. Further details regarding the image acquisition are listed in Table 2. To reduce bowel movement, glucagon (1 mg/ml, 1 ml intramuscularly) and Buscopan® (10 mg/ml, 1 ml intravenously) were administered before scanning. The Buscopan® injection was repeated before the acquisition of the dynamic images. The DME contained the information required for extraction of both T1 weighted and T2* weighted contrast enhancement curves. Two radiologists with 14 and 7 years of experience with abdominal MRI delineated the tumor region of interest on the T2w images with DW images as guidance.

**Table 2:** Overview of MR imaging parameters used in the different sequences.

Image sequence	T2w	T2*w	DW	DME
Sequence	FSE	FFE	2D EPI	3D EPI
Repetition time / s	2.82–3.04	9.49	3	0.38
Echo time / ms	80	4.6, 13.8, 23.0, 32.2, 41.4	75	4.6, 13.9, 23.2
Averages	6	3	6	1
Acquisition matrix	256/254	180/120	80/60	92/90
In plane resolution / mm	0.35	0.70	1.25	0.70
Slice thickness / mm	2.50	3.00	4.00	10
Slice separation / mm	2.75	4.00	4.30	5
Scan time^†^ / min	7	6	8	7

T2w: T2-weighted; T2*w: T2*-weighted; DW: Diffusion weighted; DME: Dynamic multi echo; FOV: field of view; FSE: fast spin echo; EPI: echo planar imaging; FFE: Steady state gradient echo; †: Median values, dependent on number of imaged slices.

### 2.3 Image pre-processing

DME images were acquired for 60+ time points. A subset of these images was selected to normalize between patients by adjusting for variation in timing of contrast agent injection and still depict the entire available temporal development. To determine this subset, images most closely matching a temporal resolution Δt of 4 s for the first eight images were selected, starting with the arrival of contrast agent, followed by six images with Δt of 80 s. Thus, in total 14 images at *t* = 0, 4, …, 24, 28, 108, 188, …, 508 s were selected.

Images from the different MRI sequences (T2w, T2*w, DW and DME) were rigidly registered and resampled towards a common grid of 1 × 1 × 1 mm^3^ voxels. The registration was focused by using a mask on a cuboid box with a 20 mm margin around the union of both manual delineations and was further restricted to the field of view present in all images. This process aimed to imitate a user drawing an initial bounding box around the tumor. In addition, the images were cropped to this region of interest to focus on the relevant anatomy and aid in balancing the dataset in terms of tumor and non-tumor voxels. Image pre-processing was done in Python 3.7 [22], using SimpleITK 1.2 [23] and SimpleElastix 1.1 [24].

### 2.4 Machine learning

The segmentation task was treated as a two class voxelwise classification problem to separate tumor and normal tissue voxels. The performance of four algorithms was evaluated, namely linear discriminant analysis (LDA) [25], quadratic discriminant analysis (QDA) [26], support vector machines (SVM) [27] and adaptive boosting (ADA) [28]. For the T2w images, the intensity of the voxel of interest as well as its eight in-plane neighbors were used as features, sorted by their intensities. For the DW and T2*w images, features consisted of the seven diffusion b-value images or the five echo time images, respectively. The DME feature set comprised the image intensities at the 14 selected timepoints with three echoes each. To correct for inter-patient variations, the voxel intensities were normalized by calculating the z-scores within each image type and patient. The union of both manual contours was used as ground truth. The features were arranged in a data matrix as described in [29] and [30] and illustrated in Supplementary Fig. S1. The dataset contained only 8% tumor voxels. Random undersampling was used to achieve a class balance on the per patient level. Thus, for each patient, a number of non-tumor voxels were randomly chosen to match the number of tumor voxels. The analysis was performed in MATLAB® 2019a (The Mathworks, Inc., Natick, Massachusetts, USA).

### 2.5 Post-processing

The initial, automatic generated segmentation predicted by the trained model was post-processed semi-automatically before further evaluation. First, a median filter smoothed the borders of the predicted segmentation. Second, a watershed segmentation was applied to the Euclidean distance transformed mask, to separate and distinguish connected regions. Third, the identified connected regions were classified as either belonging to the tumor or otherwise discarded. In a clinical application, such a separation could be achieved by a mouse click by the user. For the presented analysis, this selection process was simulated by randomly sampling one voxel (a seed) per slice within the ground truth delineation. The final segmentation consisted only of the regions containing these seeds, while all other regions were discarded. The post-processing was implemented in Python 3.7 [22] using the SimpleITK 1.2 [23] package.

### 2.6 Performance evaluation

Leave-one-out cross validation on the patient level was used to simulate that a trained model is used to predict the tumor volume of a new patient. The Sørensen-Dice similarity coefficient (DICE) [31] was used to evaluate the agreement between the ground truth *G* and the predicted segmentation *P*, and was defined as:

$$\text{DICE}=\frac{2\left|P\cap G\right|}{\left|P\right|+\left|G\right|}$$

Mean symmetric surface distance (MSD) [32] was included as an additional distance-based measure, defined as:

$$\text{MSD}=\frac{1}{N_{G}+N_{P}}\left(\sum_{i=1}^{N_{G}}\left|d_{i}^{G\to P}\right|+\sum_{i=1}^{N_{P}}\left|d_{i}^{P\to G}\right|\right)$$

The total number of voxels in the respective surface was denoted as *N* and $d_{i}^{A\to B}$ was the minimal Euclidean distance in 3D from point *i* on surface *A* to a point on surface *B*. Results were summarized as median (MED) and interquartile range (IQR).

### 2.7 Experimental procedure

In a first step, the performance of the different algorithms (LDA, QDA, SVM and ADA) was assessed using T2w image-based features as the only input. To gauge differences in performances among the four algorithms, the Friedman test for repeated measurements [33] was used. If the test indicated a significant difference, a post-hoc, two-sided Wilcoxon signed rank test with Bonferroni correction for multiple testing was used to identify significantly different pairings. The algorithm giving the highest performance measured by DICE was selected for the further analysis.

In a second step, the potential benefit of using functional MRI-based features was investigated. The performance of mono-sequence models based on T2*w, DW or DME image features alone, and multi-sequence models based on any combination of T2w, T2*w, DW and DME was evaluated and compared with the best T2w-only model. The difference in performance was assessed using a two-sided Wilcoxon signed rank test with Bonferroni correction.

The statistical analysis was performed with a significance level of 0.05 using the python packages SciPy 1.7 [34] and scikit-posthocs 0.6 [35], and results were visualized using matplotlib 3.4 [36].

## 3 Results

**Fig. 1:**
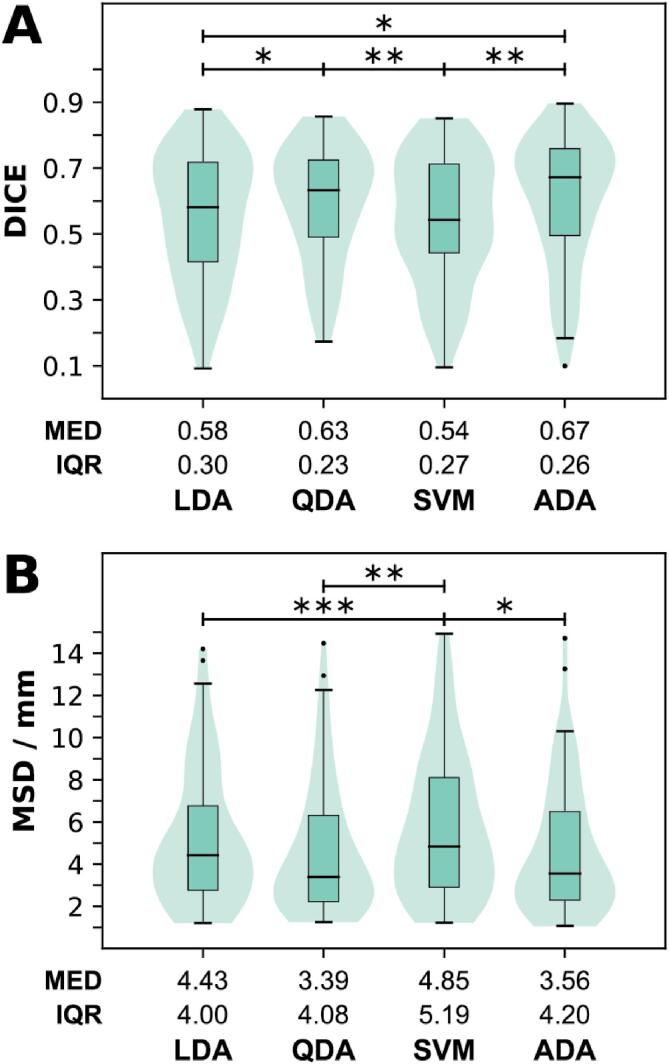
(**A**) Sørensen-Dice similarity coefficient (DICE) and (**B**) mean symmetric surface distance (MSD) visualized as combined box and violin plots. T2w image-based features were used to train models using four different algorithms (LDA: Linear discriminant analysis, QDA: Quadratic discriminant analysis, SVM: Support vector machines, ADA: Adaptive boosting). Median (MED) and interquartile range (IQR) are listed. As the Friedman test indicated a significant difference (p < 0.01 for DICE and p < 0.001 for MSD), a post hoc, two-sided Wilcoxon signed rank test was applied to all pair-wise combinations. Only significant results are indicated in the figure. (*: p < 0.05, **: p < 0.01, ***: p < 0.001).

The performance of the four algorithms LDA, QDA, SVM and ADA with the mono-sequence model based on T2w image features is shown in Fig. 1. The Friedman test was significant both for DICE (p < 0.01) and MSD (p < 0.0001). The highest (best) DICE score was achieved by the ADA-based model with median [IQR] of 0.67 [0.26] (MSD: 3.6 [4.2] mm; second best). The QDA-based model resulted in the lowest (best) MSD with 3.4 [4.1] mm (DICE: 0.63 [0.23]; second best). No significant difference was indicated between ADA and QDA by the post hoc Wilcoxon test, neither for DICE nor MSD. The lowest performance was observed for SVM (DICE: 0.54 [0.27], MSD: 4.9 [5.2] mm). This was significantly different to both ADA (DICE: p = 0.003, MSD: p = 0.03) and QDA (DICE: p = 0.005, MSD: p = 0.001). In addition, the measured MSD for SVM was also significantly different to LDA (p < 0.001). In the further analysis, only ADA was used, since it gave the best segmentation result (highest DICE) with T2w images alone.

**Fig. 2:**
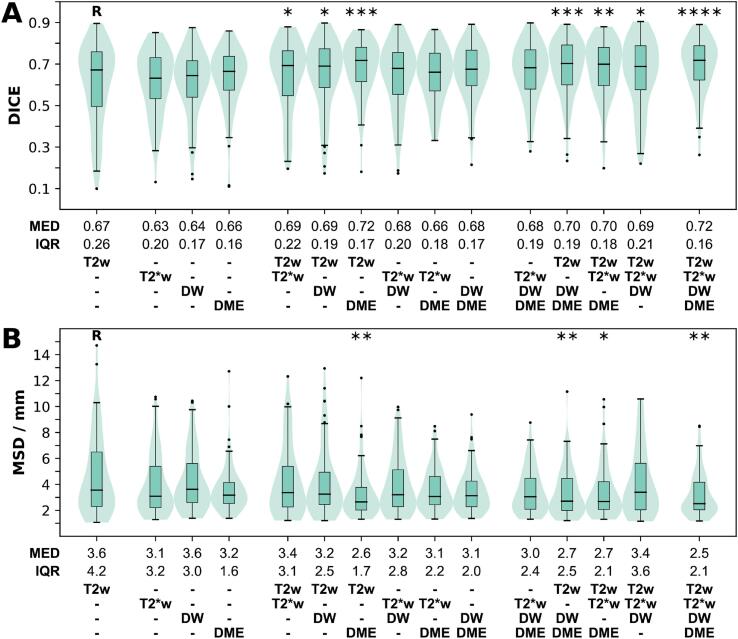
(**A**) Sørensen-Dice similarity coefficient (DICE) and (**B**) mean symmetric surface distance (MSD) visualized as combined box and violin plots. The performance is shown for mono-sequence models using features based on single image modalities (T2w: T2-weighted, T2*w: T2*-weighted, DW: diffusion weighted, DME: dynamic multi echo) as well as multi-sequence models using combinations of these feature sets. Median (MED) and interquartile range (IQR) are listed. Two-sided Wilcoxon signed rank test with Bonferroni correction was used to identify performances significantly different from the T2w feature based reference model (R). Only significant results are indicated in the figure. (*: p < 0.05, **: p < 0.01, ***: p < 0.001, ****: p < 0.0001).

Fig. 2 shows the results for models trained on the functional MRI-based features alone as well as multi-sequence combinations of the different feature sets. Mono-sequence models using T2*w, DW or DME features did not significantly improve the performance as compared to T2w alone. The same was observed for any combination of these three functional feature sets, i.e., no significant improvement was observed for combinations T2w was not part of. In contrast, all combinations of T2w with one or more functional feature sets resulted in a significantly higher DICE relative to T2w alone. Models with the four best DICE scores (>0.70) all had T2w + DME included (T2w + DME, DICE 0.72, p < 0.001; T2w + DW + DME, DICE 0.70, p < 0.001; T2w + T2*w + DME, DICE 0.70, p = 0.002; T2w + T2*w + DW + DME, DICE 0.72, p < 0.0001). The importance of T2w + DME for good segmentation was also observed when using MSD as metric; a significant improvement in MSD was only observed if T2w + DME features were included in the model (T2w + DME, MSD: 2.7 mm, p = 0.003; T2w + DW + DME, MSD: 2.7 mm, p = 0.01; T2w + T2*w + DME, MSD: 2.7 mm, p = 0.03; T2w + T2*w + DW + DME, 2.5 mm, p = 0.002).

**Fig. 3:**
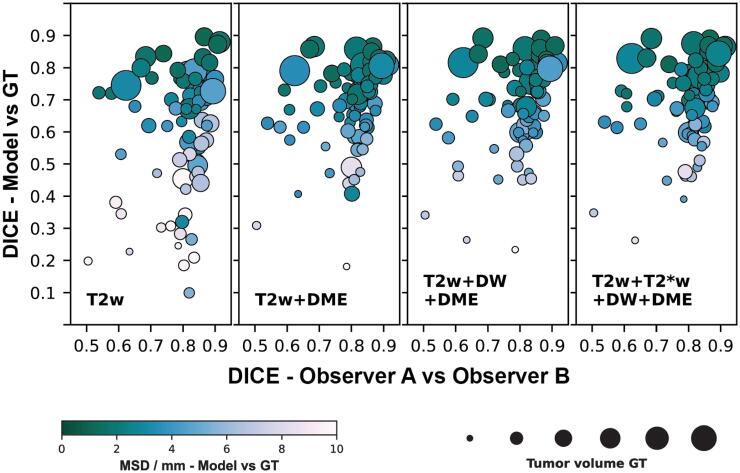
Visualization of the association between the two performance metrics, Sørensen-Dice similarity coefficient (DICE) and mean symmetric surface distance (MSD), and the interobserver DICE and the tumor volume. The median and interquartile interobserver DICE was 0.82 [0.07] with an MSD of 1.2 [0.4] mm. The panels show results of models trained using adaptive boosting (ADA) for different combinations of image features (T2w: T2-weighted, T2*w: T2*-weighted, DW: diffusion weighted, DME: dynamic multi echo, GT: ground truth).

For the two manual delineations, which formed the ground truth, the median interobserver agreement in DICE was 0.82 [0.07] with an MSD of 1.2 [0.4] mm. As indicated in Fig. 3, the DICE between the semi-automatic segmentation and ground truth seemed to be correlated to the interobserver DICE and the tumor volumes. Especially, segmentations with a low performance were more frequently observed for smaller tumors.

**Fig. 4:**
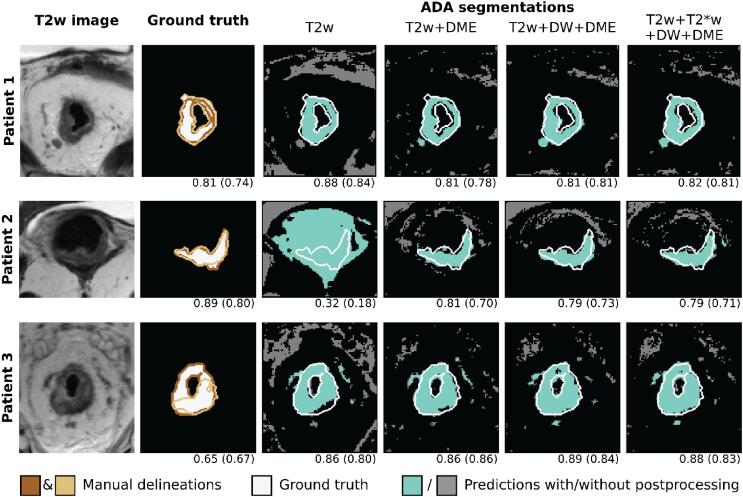
Visualization of the automatic segmentations created using adaptive boosting (ADA) models trained on different combinations of input features (T2w: T2-weighted, T2*w: T2*-weighted, DW: diffusion weighted, DME: dynamic multi echo). The T2w image and the manual delineations made by two experts are shown in addition. The numbers below each delineation or prediction state the Sørensen-Dice similarity coefficient (DICE) for the shown slice as well as the patient DICE (in parentheses).

Fig. 3 further demonstrates that the performance measured by DICE and MSD was quite stable when comparing the segmentations made by models based on T2w + DME, T2w + DW + DME and T2w + T*2w + DW + DME feature sets. Thus, for most patients, there is little variation in the measured performance both in DICE and MSD. This stability can also be appreciated in Fig. 4, where the generated segmentations are shown for three patients. Moreover, the figure illustrates that T2w features alone were in some cases not sufficient to adequately predict the tumor volume, as seen for Patient 2 in the second row. In such cases, adding functional images as input improved the segmentation result.

## 4 Discussion

In this exploratory analysis of multi–sequence MRI data from patients with rectal cancer, we showed that the performance of semi-automatic tumor volume segmentation using voxelwise classification was improved by adding functional MRI information compared to the use of anatomical T2w MRI alone. DME MRI information was found to be most valuable in this context.

Inclusion of additional images besides T2w and DME did not improve results further. The overall performance metrics for T2w + DME feature based models (DICE: 0.72 [0.17], MSD 2.7 [1.7] mm) were comparable to models based on the combination of all four feature sets (DICE: 0.72 [0.16], MSD: 2.5 [2.1] mm). This observation was further supported by the stability of the performance for individual patients shown in Fig. 3, Fig. 4. DME MRI is not part of the current clinical routine for rectal cancer, but our investigation shows it may add useful information for the purpose of tumor detection.

The ADA algorithm seemed to have the flexibility needed in this classification problem. It outperformed more rigid algorithms such as LDA. However, as illustrated in Fig. 4, the automatic voxelwise classification approach needed to be combined with a semi-automatic post-processing step to achieve good results. The implemented post-processing required seeds, which in this analysis were randomly and automatically set within the (known) tumor volume. In practice, such seeds could be set by an expert by simply clicking on the image and performance could further be improved e.g., by setting multiple seeds per slice, or by eroding marginal connections and unwanted regions. As seed selection only requires a few clicks, the process would still reduce the workload and time investment compared to full manual delineation. Such a machine learning-assisted workflow is highly relevant for MRI-guided radiotherapy using the hybrid MRI-Linac, where automatic or semi-automatic segmentation of the target and organs at risk is expected to mitigate the time- and labor-intensive tasks of manual contour delineation, and at the same time reduce inter- and intraobserver variability in contour delineation [37]. Integration of automatic or semi-automatic segmentation may provide the possibility for fast inter- and intrafraction radiotherapy adaptation, and also automatic calculation of dose accumulation. Overall, this promises greater precision and personalization of radiotherapy.

When comparing the segmentation performance achieved in this paper to previously published classical machine learning techniques, we observed similar performances for comparable input images. One example is Irving et al., where DCE MRI-based pieces of parts supervoxel segmentation achieved a median DICE of 0.63 relative to the manual delineation [5]. The closest match in our analysis was the ADA model trained only on the DME feature set, giving a median DICE of 0.66. Heeswijk et al. used an automatic region growing approach based on DW b1000/b1100 images [17], achieving a mean DICE of 0.68 ± 0.15. In our analysis, the DW-feature-based ADA model resulted in a mean DICE of 0.61 ± 0.16 (MED [IQR]: 0.64 [0.17]). However, in contrast to the other studies, we showed that segmentation results could be improved by combining both anatomical and functional MRI information improved the segmentation results, this was not evaluated in the other studies.

Deep learning techniques, like neural nets, have shown promising results for automatic tumor segmentation. Trebeschi et al. used a convolutional neural network (CNN) with T2w and DW images to segment locally advanced rectal cancer and achieved a mean DICE of 0.68 ± 0.07 and 0.70 ± 0.07 compared to two manual readers, respectively [4]. The T2w and DW-based model presented in our current work resulted in a median DICE of 0.69 [0.19]. This showed that classical machine learning approaches should not be disregarded, even though neural net techniques may achieve a more stable segmentation with less variation between patients.

The patient cohort analyzed in this study was also the basis for training a 2D U-Net for automatic segmentation [38]. In [38], the use of T2w images alone resulted in a DICE of 0.77 [0.21] and T2w + DW images in a DICE of 0.76 [0.18] for patients in a holdout test set. Thus, adding DW images did not improve the U-Net segmentation which stands in contrast to the slight improvement in DICE observed in the present analysis for T2w relative to T2w + DW based models. This suggested that the classical machine learning methods may benefit more than deep learning-based models by the inclusion of functional MRI data. The DICE for the U-Net results were higher than those of the ADA model in our current study (0.77 versus 0.72 for best cases). However, as different subsets of the cohort were used in the training due to availability of image data, this direct comparison should be treated with due caution. As the focus of the present analysis was to systematically study the influence of different functional MRI sequences in many combinations, a classical machine learning approach was chosen. Not only was it more computationally manageable, but it was also better suited for a smaller patient cohort as it operated on the voxel level.

The union of two manual delineations was used as ground truth in this study. The real extent of the tumor would need to be determined histologically and experts’ delineations are the best available approximation. Using the union instead of an individual contour represented a conservative approach that aimed at including all suspicious tissue in the ground truth. For manual delineations, the delineated volume depends not only on the observer but also on the available MR images, as noted by Hearn et al. [6]. The interobserver agreement in the underlying dataset of our study was determined as DICE of 0.82 [0.07] with an MSD of 1.2 [0.4] mm. This was in good agreement with variations reported in previous studies [4], [5], [6]. Comparing the interobserver DICE to the DICE of our best performing model (0.72) suggested that the model may not be sufficient on its own. However, it could still have value as a contouring support tool and for reducing the time used in the overall workflow. Such a support tool could provide a suggested initial segmentation fast. After reviewing the suggestion, the user could then accept or alter the segmentation.

In the current analysis, DICE and MSD were used as performance measures. Both capture different aspects of the performance but also have their specific drawbacks [39]. One example is the volume dependency of the DICE metric, as for small structures, variation in single voxels can result in large changes to the measured performance. This effect could contribute to the low performance observed for small tumors in Fig. 3.

As this study was based on data from a single center, an extension with data from different centers would be beneficial. This could eliminate biases in the training data, which would in turn improve the generalization of the model. It could also be beneficial to include manual delineations made by experts from other centers or to use consensus delineations for the training.

In conclusion, semi-automatic segmentation of rectal cancer improved when machine learning models were trained with a combination of T2w and functional MRI data. The best results were obtained when both T2w and DME features were included in the model. Since contrast enhanced MRI currently is not part of routine diagnostic MRI in rectal cancer, further studies are needed to determine if it should be added to future MRI protocols.

## Declaration of Competing Interest

The authors declare that they have no known competing financial interests or personal relationships that could have appeared to influence the work reported in this paper.
