# Supplementary material for: Semi-automatic tumor segmentation of rectal cancer based on functional magnetic resonance imaging
Source: Phys Imaging Radiat Oncol. 2022 May 11;22:77–84. doi: 10.1016/j.phro.2022.05.001 (PMC9114680; doi:10.1016/j.phro.2022.05.001)

## Supplementary Figure S1

Illustration of image feature extraction and organization into the data matrix

(T2w: T2-weighted, T2*w: T2*-weighted, DW: diffusion weighted,

DME: dynamic multi echo; TE: Echo time; b: Diffusion b-value)

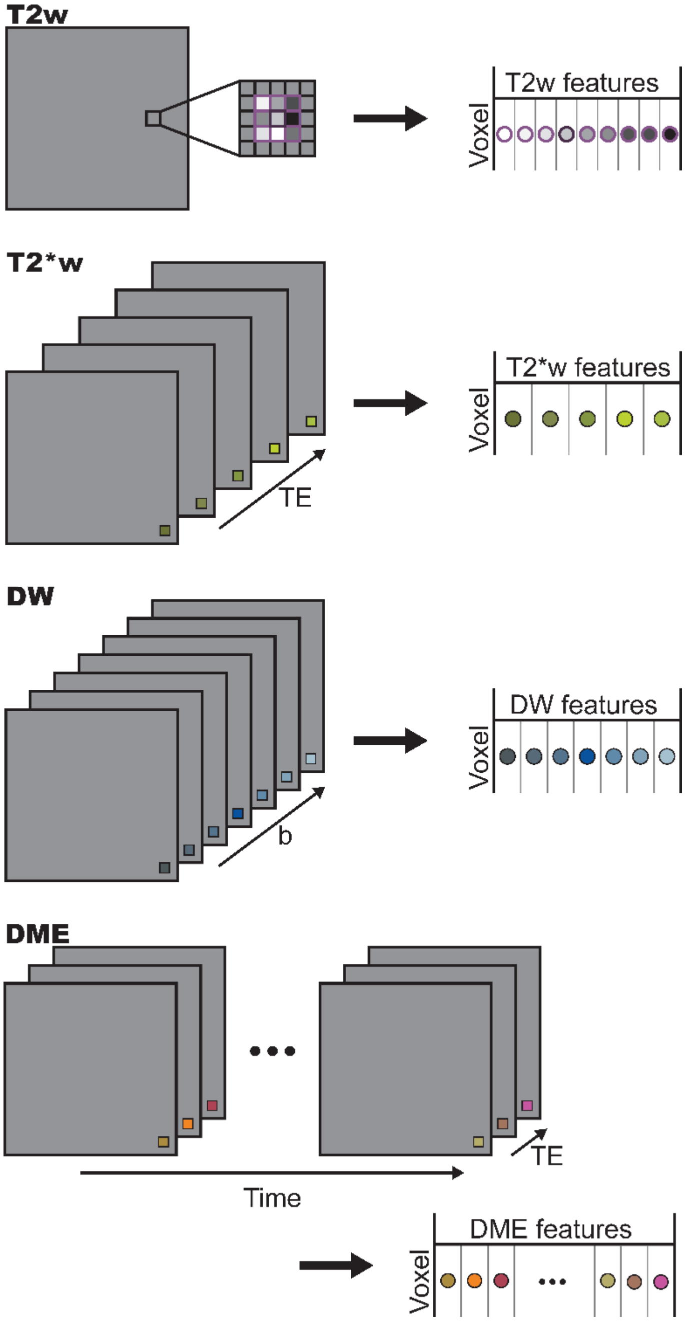

Supplement: Supplementary data 1 [file mmc1.pdf]
